# Neuromodulation in acute traumatic brain injury: a tool in the rehabilitation process that needs to be investigated

**DOI:** 10.1590/1516-3180.2021.0988.11052022

**Published:** 2022-09-23

**Affiliations:** I PhD. Associate Professor, Laboratory of Neurorehabilitation and Neuromodulation, Department of Physiological Sciences, Universidade Federal do Espírito Santo (UFES), Vitória (ES), Brazil; Associate Professor, Department of Integrated Health Education, Physical Therapy Course, Universidade Federal do Espírito Santo (UFES), Vitória (ES), Brazil.; Universidade Federal do Espírito Santo, Department of Integrated Health Education, Vitória, ES, Brazil; II PT, MSc. Physiotherapist, Laboratory of Neurorehabilitation and Neuromodulation, Department of Physiological Sciences, Universidade Federal do Espírito Santo (UFES), Vitória (ES), Brazil.; III MD, PhD. Professor, Neurosurgery Division, Department of Neurology, Clinical Hospital, Faculdade de Medicina da Universidade de São Paulo (FMUSP), São Paulo (SP), Brazil.

Dear Editor,

In 2014, while one of us (FZSA) was working towards his doctorate, he had the opportunity to follow about 200 patients who had suffered severe head trauma (traumatic brain injury, TBI), from the first day after the trauma until one year later. In relation to these cases, a variety of hypothetical questions arose: Could noninvasive brain stimulation (NIBS) help to contain the advancement of neurotoxicity, help with reorganization after brain injury and optimize brain neuroplasticity? In addition to the question of whether it was effective, would it be safe? What would be the barriers?

At that time, studies investigating the effects of NIBS in patients suffering from TBI had been conducted,^1–3^ but none of them related to acute patients.

So far, little progress has been made in neuromodulation studies on this type of patient.^4^ In fact, neuromodulation is a consolidated form of treatment for various neurological problems consequent to diseases such as depression, stroke, pain and others.^5^ Neurophysiological effects are known in the literature, although some questions about mechanisms exist in relation to some diseases.^6^

However, various questions remain to be addressed in relation to TBI. This condition is a major public health problem worldwide and there is a need to move forward regarding its treatment. Neuromodulation may therefore form an important tool in the rehabilitation process for this disease condition.^7^

Many barriers exist in relation to how NIBS is performed in critically ill patients, especially with regard to transcranial direct current stimulation (TDCS) and transcranial magnetic stimulation (TMS). The issues involved include safety, clinical instability, extent of the injury, unfavorable hospital environment, heterogeneity of the injuries of TBI patients and treatment adherence after hospital discharge.^8^ One interesting study showed good results from use of NIBS among patients with acute stroke and, although each condition has its specific characteristic, that study highlights the potential for use of NIBS among patients with acute brain injury in general.^1^

One of the great challenges in proposing clinical trials to test use of NIBS among patients with acute TBI concerns safety, considering that these are patients with great clinical instability. Thus, neurological stability needs to be ensured, given that patients with acute brain injury have high incidence of epilepsy, for example.^2^ Another great challenge for researchers in preparing the study design is to fit the protocols to the sample homogeneity, considering that TBI cases are complex and have different characteristics. These complexities and differences form a great barrier to applying pre-established protocols such patients. Hence, patients' individuality needs to be respected and application of protocols and assemblies of equipment close to the target have to be guided by very careful evaluation. Use of tools such as functional magnetic resonance imaging, electroencephalograms, positron emission tomography (PET) scans and neuronavigation may perhaps be essential.^2^

Although results are only available from a few studies, methodologically well-designed works with good numbers of patients and with follow-ups need to be envisaged, so that not only can the effects of stimulation be identified over the short term, but also it can be known whether the effects persist. Identification of clinical predictors to identify possible impacts of acute-phase variables on the outcomes of patients undergoing NIBS can also be suggested. In this way, it can be ensured that subjecting patients to stimulation retains a good cost-benefit relationship and is safe.

A wide range of measurements of effects is required, given that the sequelae of patient suffering from TBI have a wide spectrum. Therefore, motor, cognitive, psychiatric, functional and quality-of-life factors need to be assessed, without neglecting the patients' biopsychosocial characteristics. Among these measurements of effects, it can also be suggested that brain injury and recovery should be evaluated through biomarkers. This can strengthen the biological plausibility of the effects and be correlated with patients' clinical and functional improvements.

However, answers regarding the effects of NIBS on acute patients with head trauma over the short, medium and long terms are far from being obtained. The uncertainties are compounded by difficulties in designing and conducting a robust clinical trial. One interesting path would be to elaborate a feasibility study to identify barriers and facilitators regarding this approach among this type of patients. This could form an important study that would help in shaping the most appropriate methodology for clinical trials and even help in decision-making and clinical care for these patients.

## References

[1] Bender Pape TL, Herrold AA, Guernon A, Aaronson A, Rosenow JM (2020). Neuromodulatory Interventions for Traumatic Brain Injury. J Head Trauma Rehabil.

[2] Demirtas-Tatlidede A, Vahabzadeh-Hagh AM, Bernabeu M, Tormos JM, Pascual-Leone A (2012). Noninvasive brain stimulation in traumatic brain injury. J Head Trauma Rehabil.

[3] Maas AIR, Menon DK, Adelson PD (2017). Traumatic brain injury: integrated approaches to improve prevention, clinical care, and research. Lancet Neurol.

[4] Pruski A, Cantarero G (2020). Transcranial Direct Current Stimulation for Motor Recovery Following Brain Injury. Curr Phys Med Rehabil Rep.

[5] Li S, Zaninotto AL, Neville IS (2015). Clinical utility of brain stimulation modalities following traumatic brain injury: current evidence. Neuropsychiatr Dis Treat.

[6] Leśniak M, Polanowska K, Seniów J, Czlonkowska A (2014). Effects of repeated anodal tDCS coupled with cognitive training for patients with severe traumatic brain injury: a pilot randomized controlled trial. J Head Trauma Rehabil.

[7] Straudi S, Bonsangue V, Mele S (2019). Bilateral M1 anodal transcranial direct current stimulation in post traumatic chronic minimally conscious state: a pilot EEG-tDCS study. Brain Inj.

[8] Areas FZ, Schwarzbold ML, Diaz AP (2019). Predictors of Hospital Mortality and the Related Burden of Disease in Severe Traumatic Brain Injury: A Prospective Multicentric Study in Brazil. Front Neurol.

